# Correlation between proteinuria and cardiac biomarker levels in kidney transplant recipients

**DOI:** 10.1080/0886022X.2026.2731733

**Published:** 2026-11-10

**Authors:** Boglárka Bujáki, Taisiia Ovcharova, Marianna Pócsi, Béla Nagy Jr., Tamás Bence Pintér, Attila Borbély, Miklós Fagyas, József Balla, Réka P. Szabó

**Affiliations:** a Department of Nephrology, Faculty of Medicine, Institute of Internal Medicine, University of Debrecen, Debrecen, Hungary; b Department of Laboratory Medicine, Faculty of Medicine, University of Debrecen, Debrecen, Hungary; c Faculty of Medicine, Institute of Cardiology, University of Debrecen, Debrecen, Hungary

**Keywords:** Kidney transplantation, proteinuria, IL-33, soluble ST2, cardiovascular risk, graft dysfunction

## Abstract

Kidney transplantation is the preferred treatment for end-stage kidney disease, with improved short-term outcomes; however, long-term graft and patient survival remain suboptimal. Proteinuria is a strong predictor of adverse graft and cardiovascular outcomes. We investigated the relationship between proteinuria and selected cardiac and inflammatory biomarkers, including the interleukin-33 (IL-33)/soluble ST2 (sST2) axis, malondialdehyde, neprilysin, growth differentiation factor-15 (GDF-15), and angiotensin-converting enzyme 2 (ACE2), in kidney transplant recipients, with a focus on patients with heart failure. Methods: Sixty-five transplant recipients were included: 15 kidney transplant recipients with heart failure and 49 controls consisting of kidney or heart transplant recipients without heart failure. Patients were followed between November 2023 and June 2024 at the Division of Nephrology at the University of Debrecen. Proteinuria was assessed using the urinary protein-to-creatinine ratio. Laboratory analyses included routine biochemical parameters, inflammatory markers, NT-proBNP, soluble IL-33 receptor (sST2), neprilysin, GDF-15, and ACE2 activity. Discrete data were described as percentages and analyzed with Fisher’s exact test. Group comparisons were performed using the Kruskal–Wallis test, and correlations were assessed using non-parametric methods. Across all study groups, urinary protein excretion correlated weakly positively with sST2 levels (*r* = 0.27, *p* = 0.0448). Patients with heart failure had more pronounced anemia (*p* = 0.0470) and lower serum iron levels (*p* = 0.0041). Proteinuria was weakly positively associated with circulating sST2 concentrations. Further studies are needed to clarify the prognostic significance of this pathway in kidney transplant outcomes.

## Introduction

A kidney transplant is a cure for end-stage kidney disease. While short-term renal transplant success has improved over the past two decades, the rate of improvement in long-term graft and patient survival has lagged behind. Patients undergoing transplantation age are increasing, they have more comorbidities, and the most common causes of death are cardiovascular, infectious and malignant diseases [1,2]. With the huge gap between the shortage of donor organs and the number of patients on waiting lists, we must use all means to ensure the longest possible patient and graft survival for our patients. Screening for cardiovascular disease prior to kidney transplantation is warranted, since Foley’s analysis, cardiac mortality in dialysis patients is known to be 10 to 20 times higher than in the general population. After successful kidney transplantation, cardiac mortality can be reduced to a fraction, but it is still 3–5 times higher than in the average population [3–10].

One of the emerging key players in the ‘classical’ RAS (Renin-Angiotensin) axis is the enzyme chymase, a chymotrypsin-like serine protease secreted by degranulated mast cells in response to tissue injury and also present in other cells, such as cardiomyocytes. Chymase is the most efficient Ang II–synthesizing enzyme and can directly stimulate profibrotic signaling pathways, including transforming growth factor–β and matrix metalloproteinases thereby affecting tissue remodeling in many renal pathologies, including CKD (chronic kidney disease), polycystic kidney disease, diabetic kidney disease and kidney transplant rejection [11]. Therefore, selective chymase inhibition alone or in addition to conventional RAS blockade could augment the clinical efficacy of RAS inhibition to decrease the risk of cardiovascular morbidity and mortality, particularly in conditions associated with fibrotic tissue damage such as CKD, chronic allograft damage, or after myocardial infarction. Chymase can activate transforming growth factor–β1 independent of Ang II [12], which is reported to be a central factor in renal fibrosis [11,12]. This underscores the potential of selective chymase inhibition as an attractive therapeutic strategy to interfere with ACE-independent Ang II production and Ang-independent profibrotic processes.

Proteinura is fairly common immediately after transplantation, and may originate from the native kidney or the allograft, making differential diagnosis difficult. On the other hand, the persistence or late occurrence of proteinuria is indicative of graft injury. In our study, we explored whether proteinuria is associated with circulating cardio-renal biomarker levels to understand cardiovascular risk mechanisms in patients with kidney transplants. This might help us detect increased cardiovascular risk earlier in patients in the future. The biomarkers in our research were originally studied in heart disease, but biologically, they belong to the shared cardio-renal axis. This means that they may reflect not only cardiac conditions, but also renal injury and systemic [13] cardio-renal stress caused by renal injury. Through these shared pathways, kidney disease and cardiovascular disease can be closely interconnected. The idea is that kidney-transplant recipients have a high cardiovascular risk, and several pathways connect kidney injury to cardiovascular disease. Neprilysin is an enzyme that cleaves certain peptides, including natriuretic peptides (such as ANP (atrial natiruretic peptide) and BNP (brain natriuretic peptide)), which help regulate blood pressure, fluid balance, and heart function. In kidney transplantation, neprilysin plays an important role because kidney dysfunction or rejection can lead to fluid imbalance and elevated blood pressure, which can negatively impact cardiovascular health [14,15]. Soluble ST2 is a circulating biomarker from the IL-33/ST2 signaling pathway. It reflects myocardial stress, inflammation, and fibrosis. Unlike BNP/NT-proBNP, sST2 is minimally affected by kidney function, which makes it especially useful in patients with CKD and kidney transplants [16–20].

We investigated the relationship between proteinuria, a known predictor of graft outcomes and several cardiac biomarkers (IL-33, malondialdehyde, neprilysin, GDF-15, ACE2) in a subset of kidney transplant patients with heart failure [12,15,19,20]. Kidney disease and heart disease are closely related processes. In individuals with CKD, the risk of heart disease is many times higher, which can lead to worsening of their own kidney disease. The main goal of our study is to find an explanation for the increased cardiovascular risk among kidney transplant recipients, to recognize them as soon as possible and, if possible, to treat them. The applicability of markers used in clinical practice is limited, which may reduce their diagnostic accuracy [19–22]. The overall goal of our study was to explore mechanisms that may contribute to the increased cardiovascular risk in kidney transplant recipients. We examined how these biomarkers relate to eGFR to determine whether their levels are driven solely by reduced filtration or reflect independent biological pathways. Second, we compared KT-only versus KT+HF to test whether these biomarkers are heart-failure–specific or whether they can also be elevated in KT-only patients, reflecting cardio-renal stress that may contribute to increased cardiovascular risk after transplantation. For the third aim, we evaluated whether proteinuria – an established predictor of graft outcomes and cardiovascular risk – is associated with our biomarker panel. If proteinuria correlates with these biomarkers, it would support a biological link between renal injury and systemic cardio-renal stress pathways that may contribute to cardiovascular risk after transplantation. Finally, as our fourth aim, we examined how the biomarker panel relates to oxidative stress markers, as oxidative stress is also an important pathway contributing to cardiovascular risk after transplantation [23–25].

## Method

This study was performed in the Department of Nephrology at the University of Debrecen. The study included transplant patients, 15 kidney transplant recipients with heart failure (and hospitalization needed due to cardiac decompensation) and 49 controls with KT or combined KT and heart transplant without heart failure. All subjects received post-transplant care between November 2023 and June 2024. Exclusion criteria included anti-rejection treatment within 6 months, acute bacterial or viral infection, malignant disease requiring hospitalization, active oncological treatment, and pregnancy.

The main immunosuppressive regimen consisted of tacrolimus, mycophenolic acid, and steroids. Proteinuria was considered and categorized as mild when greater than 150 mg/24. Discrete data were described as percentages and analyzed with Fisher’s exact test. Continuous variables were described as mean ± standard deviation when normal, and median with 25th to 75th percentile when non-normal. For statistical analyses, the Mann-Whitney test, the Kruskal-Wallis test, the t-test, or analysis of variance was used. Proteinuria was categorized as moderate when in the range of 500 to 1000 mg/24 h, and as high when severely increased (≥ 1000 mg/24 h).

### ST2/il-33-R immunoassay

The Quantikine^™^ Human ST2/IL-33 R Immunoassay is a 4.5-h solid-phase ELISA designed to measure human ST2 in cell culture supernates, serum, and plasma. It contains recombinant human ST2 expressed in NS0 cells and has been shown to accurately quantify the recombinant factor. Results obtained with natural human ST2 showed linear curves parallel to those obtained with the Quantikine kit standards. These results indicate that this kit can be used to determine relative mass values for naturally occurring human ST2.

Sample collection: Blood samples were collected using standard aseptic techniques. It was unknown whether these patients had been taking ACE inhibitors during the measurements. Blood samples were kept at room temperature for no more than 60 min, after which the serum was separated by centrifugation at 1500 g for 15 min and stored at −20 °C until used for the experiments. Other sources of ACE used were FBS and recombinant ACE expressed in a Baculovirus expression system.

We aimed to develop a chromogenic kinetic assay to measure ACE activity using multiple enzyme sources. Our substrate of choice was FAPGG (N-[3-(2-furyl)acryloyl]-L-phenylalanyl glycylglycine; Sigma-Aldrich). The hydrolysis of FAPGG into FAP and GG by ACE represents the enzyme activity in the sample being tested. Other potential substrates included hippuryl histidyl leucine (HHL) and 1-[N-[N-(2-Aminobenzoyl)glycyl]-4-nitro-L-phenylalanyl]-L-proline, a fluorogenic substrate. HHL yields hippuric acid upon enzymatic hydrolysis, and the fluorogenic substrate yields the fluorescent product o-aminobenzoylglycine. FAPGG has been shown to be a better-suited substrate than HHL for the chromogenic assay and was used as our preferred synthetic substrate for subsequent chromogenic enzyme activity assays [26].

Routinely available laboratory serum tests were performed at the Department of Laboratory Medicine, University of Debrecen. Serum samples were stored at −70 °C; serum ACE2 activity was measured using a specific quenched fluorescent substrate (obtained from http://peptide2.com) as reported earlier [27,28].

Our final assay reagent consisted of 0.5 mM FAPGG, 500 mM NaCl, and 250 mM Tris, pH 8.25. The changes in absorbance were recorded at 340 nm. Measurements were performed using a COBAS INTEGRA 400 plus chemical analyzer or manually pipetted and measured with a CLARIOstar Plus multifunctional plate reader. The inhibitory potential of Captopril and Lisinopril was measured by adding the drugs to the measurement solution and initiating the reactions by adding the ACE [23].

## Results

Table 1 summarizes the observed ranges of unlabeled numeric variables of our study.

**Table 1 .** Summary of variables.

Variable	Kidney transplant patients with heart failure (n = 15)	Control: heart transplant patients (n = 6)	Control: kidney transplant patients without heart failure (n = 43)	*p*-value (Kruskal–Wallis test)
**Age (years)**				**0.0001**
Min	18	30	34	
Max	71	65	80	
IQR	9	22	11	
**eGFR (mL/min/1.73 m^2^)**				0.1322
Min	19	45	10	
Max	90	89	90	
IQR	41	36	35	
**Total protein/creatinine ratio (TP/Crea) (mg/mmol)**				**0.0215**
Min	7	0	1	
Max	269	7	177	
IQR	34	3	19	
**Dialysis vintage time (months)**				**0.0102**
Min	0	0	0	
Max	132	0	108	
IQR	—	—	—	
**Time after transplantation (months)**				**0.0099**
Min	1	1	5	
Max	20	14	24	
IQR	11	1	9	
**LDH (U/L)**				**0.0043**
Min	145	152	132	
Max	225	546	311	
IQR	26	266	39	
**GGT (U/L)**				0.5442
Min	15	11	11	
Max	161	146	168	
IQR	32	49	22	
**NT-proBNP**				**0.0003**
Min	119	115	9	
Max	13,932	30,297	2,793	
IQR	2,665	341	439	
**EF (%)**				**0.0007**
Min	30	58	48	
Max	70	65	69	
IQR	14	5	5	
**TAPSE (mm)**				**0.0030**
Min	14	8	14	
Max	27	20	30	
IQR	8	6	7	
**Septum (mm)**				0.4026
Min	10	7	10	
Max	15	14	16	
IQR	2	2	2	
**ACE2 activity (mU/L)**				**0.0302**
Min	16.417	5.680	11.68	
Max	62.00	55.84	97.30	
IQR	15.535	27.70	8.39	
**NEP activity (U/L)**				0.9129
Min	4.979	0.52	2.207	
Max	28.80	32.10	34.156	
IQR	6.466	17.336	8.47	
**NEP concentration (ng/L)**				0.6086
Min	110.26	131.64	266.83	
Max	179,638	180,000	180,000	
IQR	9,259.737	22,331	35,549.64	
**GDF-15 (ng/L)**				0.2111
Min	749.35	475.2847	321.7035	
Max	4,735.46	3,384.453	5,731.321	
IQR	1,636.579	1,941.094	788.21	
**MDA (ng/mL)**				0.4097
Min	0.36	21.41	1.54	
Max	276.39	21.41	612.57	
IQR	83.835	0	47.05	
**sST2/IL-33 R (pg/mL)**				0.3164
Min	13,600	—	10,300	
Max	44,800	—	32,700	
IQR	12,250	—	9,100	
**Fe (µmol/L)**				**0.0041**
Min	2.41	2.8	9	
Max	26.8	11	27.6	
IQR	11.2	7.2	8.45	
**Tacrolimus level (µg/L)**				0.6347
Min	0	0	0	
Max	21.4	12	11.4	
IQR	7.2	5.2	2	
**Everolimus level (µg/L)**				**0.0091**
Min	0	0	—	
Max	5.5	5.1	—	
IQR	3	3	—	

Abbraviations: eGFR: estimaated glomerular filtration rate; LDH: lactate dehydrogenase; GGT: gamma-glutamil-tranferase; NEP: neprilysin; NT-proBNP: N-terminal pro-b-type natiruretic peptide; GDF-15: growth/differentiation factor 15; MDA: malondialdehyde; sST2: soluble interleukin 1 receptor like 1, ST2 stands for tumorgenicity 2; Fe: ferrous-iron level.

Table 2 compares biochemical, echocardiographic, and cardiovascular biomarkers across three study groups: (1) Kidney transplant recipients with heart failure, (2) Heart transplant recipients, and (3) Kidney transplant recipients without heart failure (controls). For each variable, minimum and maximum values are reported, and overall group differences were assessed using the Kruskal–Wallis test. NT-proBNP differed significantly between groups (*p* = 0.0003), with markedly higher values in both transplant groups compared with kidney transplant controls, consistent with greater cardiac stress. The left ventricular ejection fraction (LVEF) showed significant between-group differences (*p* = 0.0007). Kidney transplant recipients with heart failure had the lowest range of LVEF, while heart transplant recipients exhibited preserved systolic function. TAPSE, a marker of right ventricular systolic function, also differed significantly (*p* = 0.003), with lower values observed particularly in the heart transplant group. Serum iron levels were significantly different across groups (*p* = 0.0041), with lower levels in heart transplant recipients compared with kidney transplant controls, suggesting altered iron handling in advanced cardiac disease and after heart transplantation. ACE2 activity differed significantly between groups (*p* = 0.0302). Kidney transplant recipients with heart failure and heart transplant recipients exhibited lower minimum ACE2 activity compared with kidney transplant controls, indicating potential dysregulation of the protective ACE2/Ang-(1–7) axis in patients with cardiac disease. In contrast, neprilysin (NEP) activity (*p* = 0.91) and NEP concentration (*p* = 0.61) did not differ significantly between groups, despite wide interindividual variability. Inflammation, oxidative stress, and fibrosis markers:

**Table 2.** Cardiac biomarkers in our study population.

Variable	Kidney transplant recipients with heart failure	Heart trasnplant patients	Kidney transplant contorls	*p* value (Kruskal-Wallis)
Nt-proBNP (ng/L)	min: 119 max: 13,932	min: 115 max: 30,297	min: 9 max: 2793	0.0003
Ejection fraction (%)	min: 30 max: 70	min: 58 max: 65	min: 48 max: 69	0.0007
TAPSE (mm)	min: 14 max: 27	min: 8 max: 20	min: 14 max: 30	0.003
Serum iron (µmol/L)	min: 2.41 max: 26.8	min: 2.8 max: 11	min: 9 max: 27.6	0.0041
ACE2 activity (mU/L)	min: 16.4 max: 62	min: 5.7 max: 55.8	min: 11.7 max: 98.3	0.0302
NEP activity (IU/L)	min: 5 max: 28.8	min: 0.5 max: 32.1	min: 2.2 max: 34.2	0.91
NEP concentration (ng/L)	min: 110 max: 179,638	min: 132 max: 180,000	min: 267 max: 180,000	0.61
GDF-15 (mg/L)	min: 749 max: 4735	min: 475 max: 3384	min: 322 max: 5731	0.21
MDA (mg/mL)	min: 0.36 max: 276	min: 21.4 max: 21.4	min: 1.5 max: 613	0.41
sST2 (pg/ml)	min: 13,600 max: 44,800	min: - max: -	min: 10,300 max: 32,700	0.32

Abbreviations: TAPSE: tricuspid annular plane systolic excursion; NEP: neprilysin; GDF: growth differentiation factor 15; MDA: malondialdehide; sST2: soluble Suppression of Tumorigenicity 2.

GDF-15, MDA, and sST2 showed no statistically significant differences between groups (*p* values of 0.21, 0.41, and 0.32, respectively); sST2 was reported only in kidney transplant recipients, with wide ranges observed in both heart failure and control subgroups.

Table 2 demonstrates that traditional cardiac biomarkers and echocardiographic parameters (NT-proBNP, LVEF, TAPSE) clearly discriminate between patient groups, validating the clinical classification of heart failure status. Among novel biomarkers, ACE2 activity- but not neprilysin or sST2- shows significant group differences, supporting a potential role for ACE2 dysregulation in cardiovascular risk among kidney transplant recipients with heart failure.

The absence of significant differences in neprilysin, GDF-15, MDA, and sST2 suggests either substantial biological variability, limited statistical power, or that these markers may reflect chronic, overlapping ­cardiorenal processes rather than disease-specific states.

Figure 1 shows the following: 1a) this graph shows a scatterplot of ACE2 enzyme activity (log-transformed, measured in mU/L) plotted against GFR (glomerular filtration rate), with 64 observations. There is no significant correlation between GFR and ACE2 activity in this dataset. The trend appears nearly flat, with both the linear and empirical lines showing little deviation across the GFR range. There is no significant relationship between GFR and NEP activity. The weak correlation (*r* ≈ 0.19) and the *p*-value above 0.05 indicate that NEP activity does not vary meaningfully with GFR in this dataset. Both the linear fit and the empirical trend line are nearly flat, hinting at minimal change in NEP activity across different GFR levels. 1c) This scatterplot shows the relationship between sST2/IL33R (soluble suppression of tumorigenicity 2/interleukin-33 receptor, measured in pg/mL and log-transformed) and GFR (glomerular filtration rate). There is no significant association between GFR and sST2/IL33R levels. Both the linear and empirical trend lines are nearly flat, suggesting that variations in kidney function (GFR) do not correspond with systematic changes in sST2/IL33R concentration in this dataset. 1d) This scatterplot shows the relationship between MDA (malondialdehyde, a marker of oxidative stress) – measured in ng/mL and log-transformed – and GFR (glomerular filtration rate). This is a significant positive association between GFR and MDA levels. As GFR increases, MDA (log-transformed) tends to increase as well, suggesting that individuals with higher kidney filtration rates have higher oxidative stress marker levels. The orange empirical trend and blue regression line are consistent, both showing an upward trajectory across the GFR range. 1e) There appears to be a negative association between NEP activity and GFR – as GFR increases, NEP activity tends to decrease. However, due to the small sample size (*n* = 7) and wide confidence intervals, this result should be interpreted cautiously. The trend may indicate a potential relationship that warrants further investigation with more data. This plot shows the relationship between ACE2 activity (mU/L, log-transformed) and GFR (glomerular filtration rate). There is a weakly positive correlation between ACE2 activity and GFR – as kidney filtration rate (GFR) increases, ACE2 activity also tends to rise. The empirical and linear trends both show a clear upward trajectory. Despite the strong association, the small sample size (*n* = 7) implies that this finding should be interpreted cautiously and ideally confirmed with more data.

Figure 1. Association of ACE2 activity, NEP activity, sST2/IL33 receptor levels, malondialdehid levels and eGFR in our study population. NEP activity, ACE2 activity adjust to eGFR in heart failure subgroup in kidney transplant recipients: Owing to the small sample size, we opted for descriptive measures without statistical testing.

**Figure:**
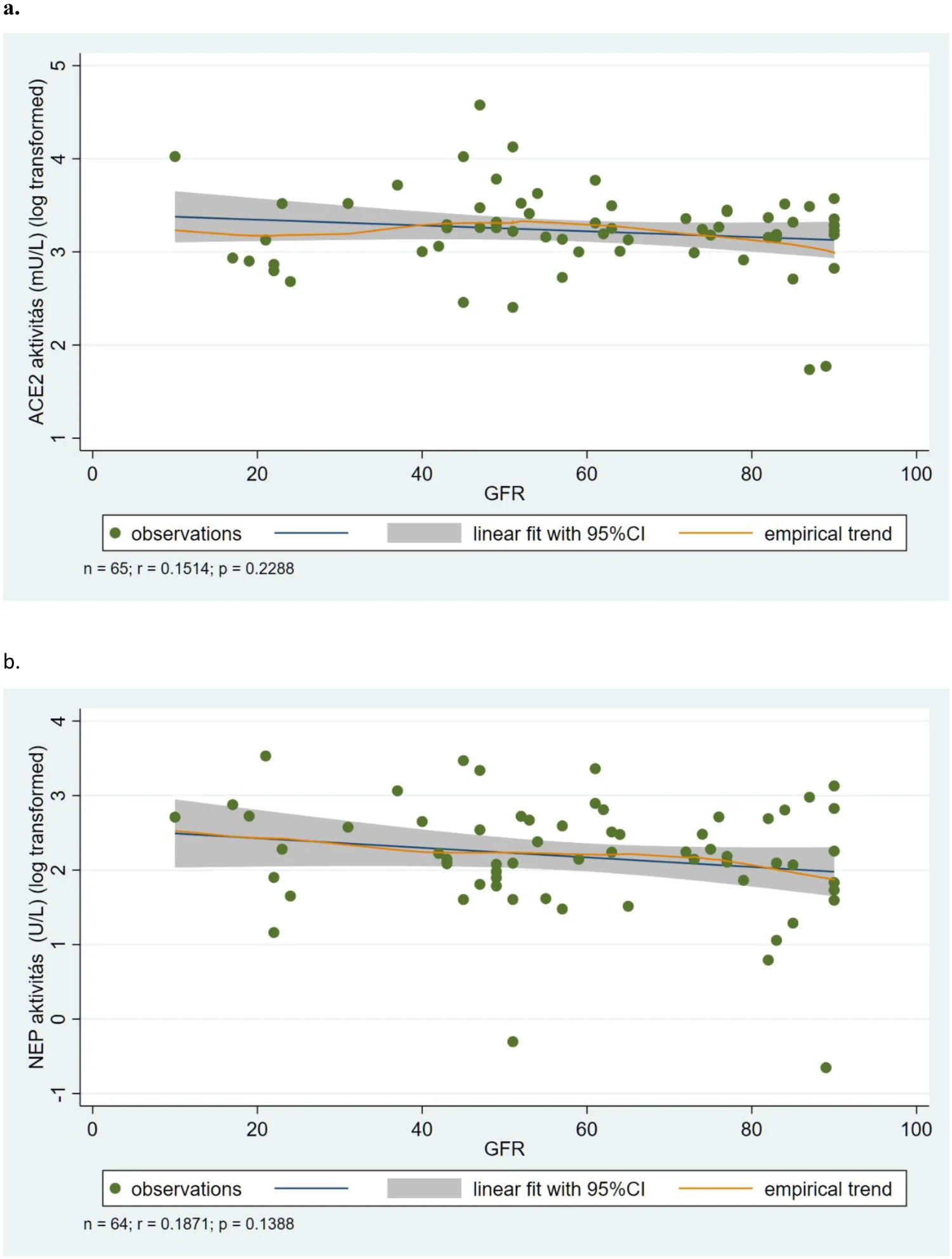

**Figure:**
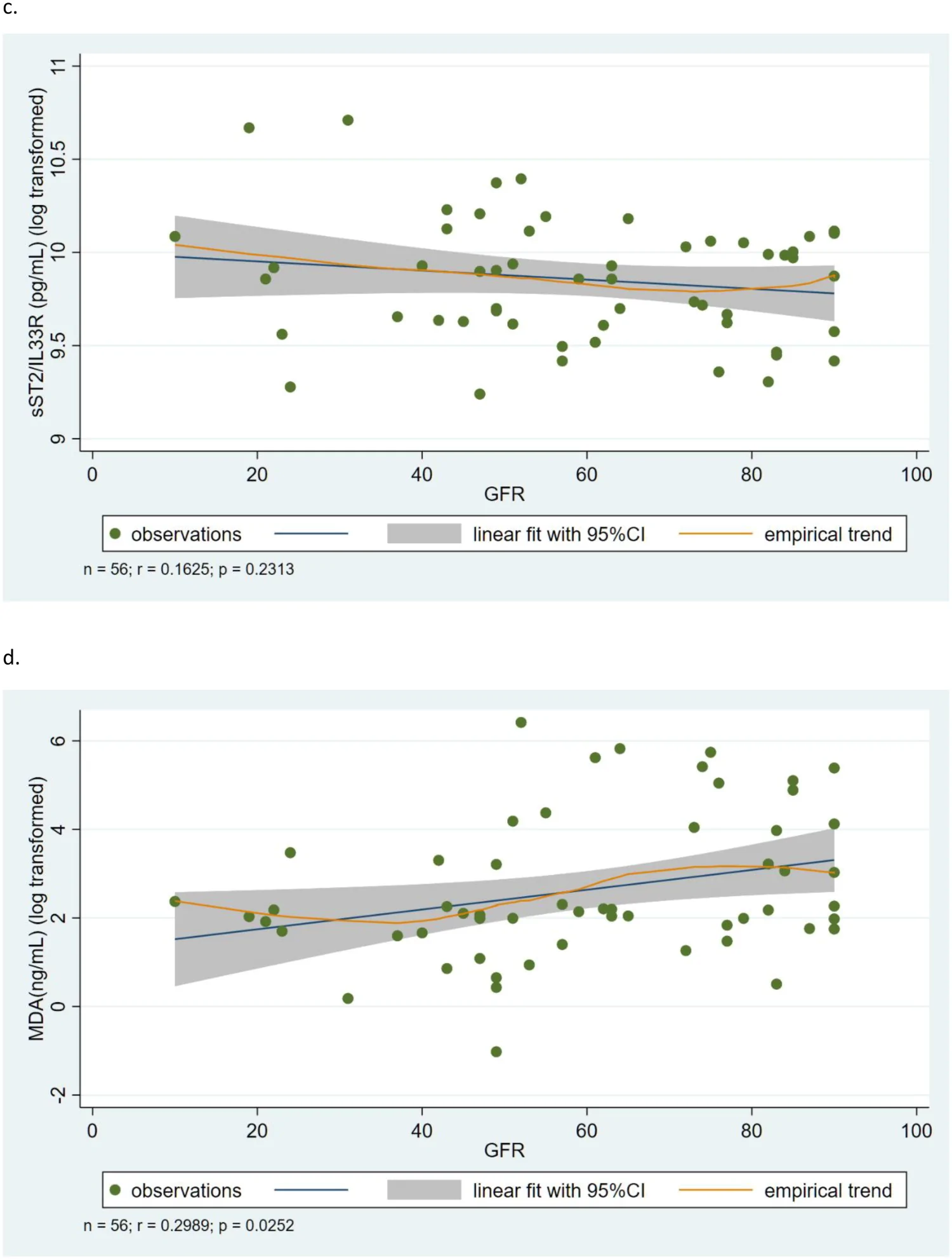

**Figure:**
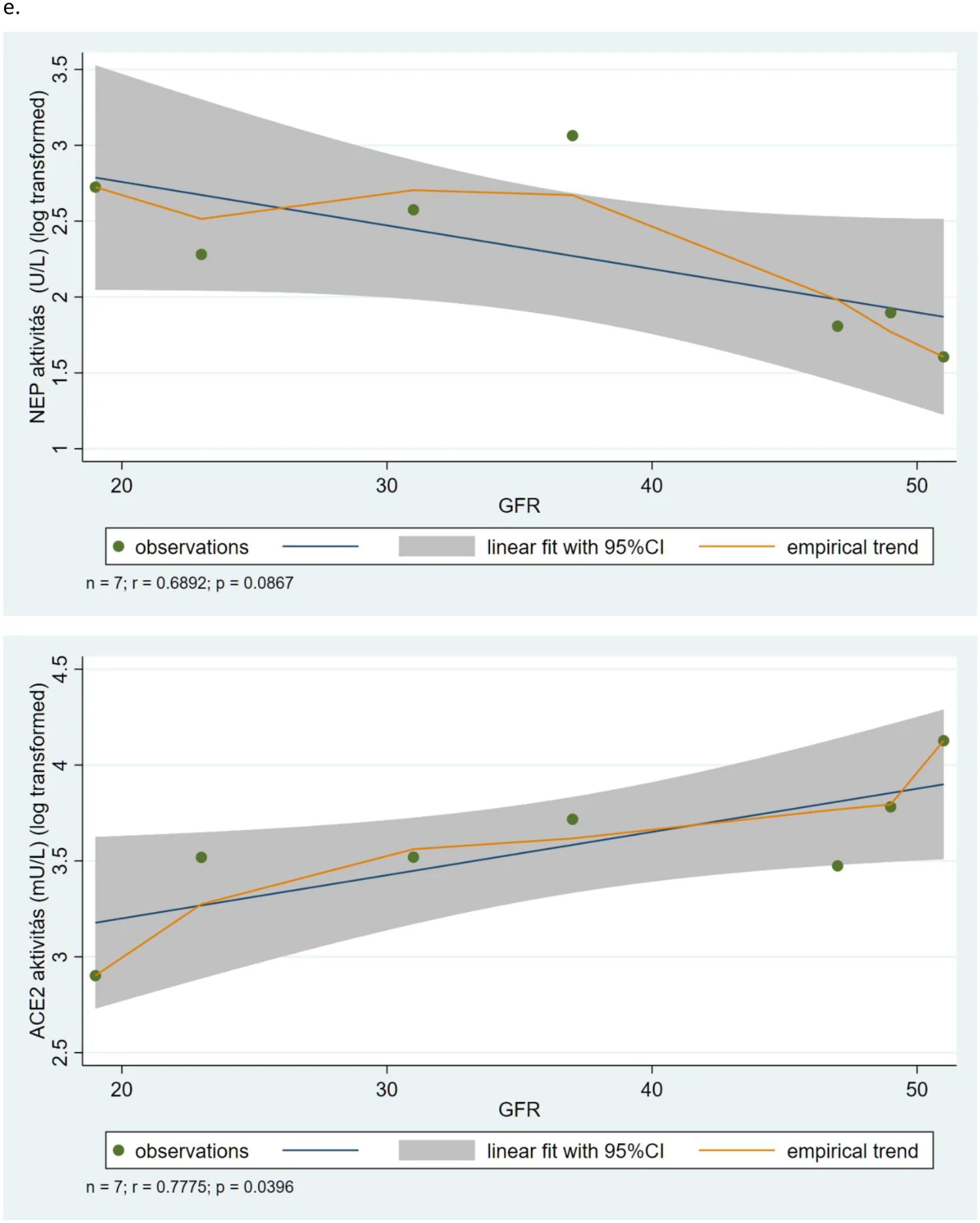

Figure 2 shows the scatterplot of relationship between sST2/IL33R (pg/mL, log-transformed) and tp/k (log-transformed), where tp/k likely represents a parameter related to protein clearance or kidney function. *r* = 0.2717: Indicates a moderate positive correlation between sST2/IL33R and tp/k. *p* = 0.0448: Statistically significant (*p* < 0.05), suggesting the correlation is unlikely due to random variation. There is a weak positive association between sST2/IL33R and tp/k – as tp/k increases, sST2/IL33R levels also tend to rise. Both the linear and empirical trend lines show an upward pattern, indicating that higher tp/k values correspond with higher circulating sST2/IL33R concentrations. The relationship is moderate in strength but statistically meaningful.

**Figure 2.**
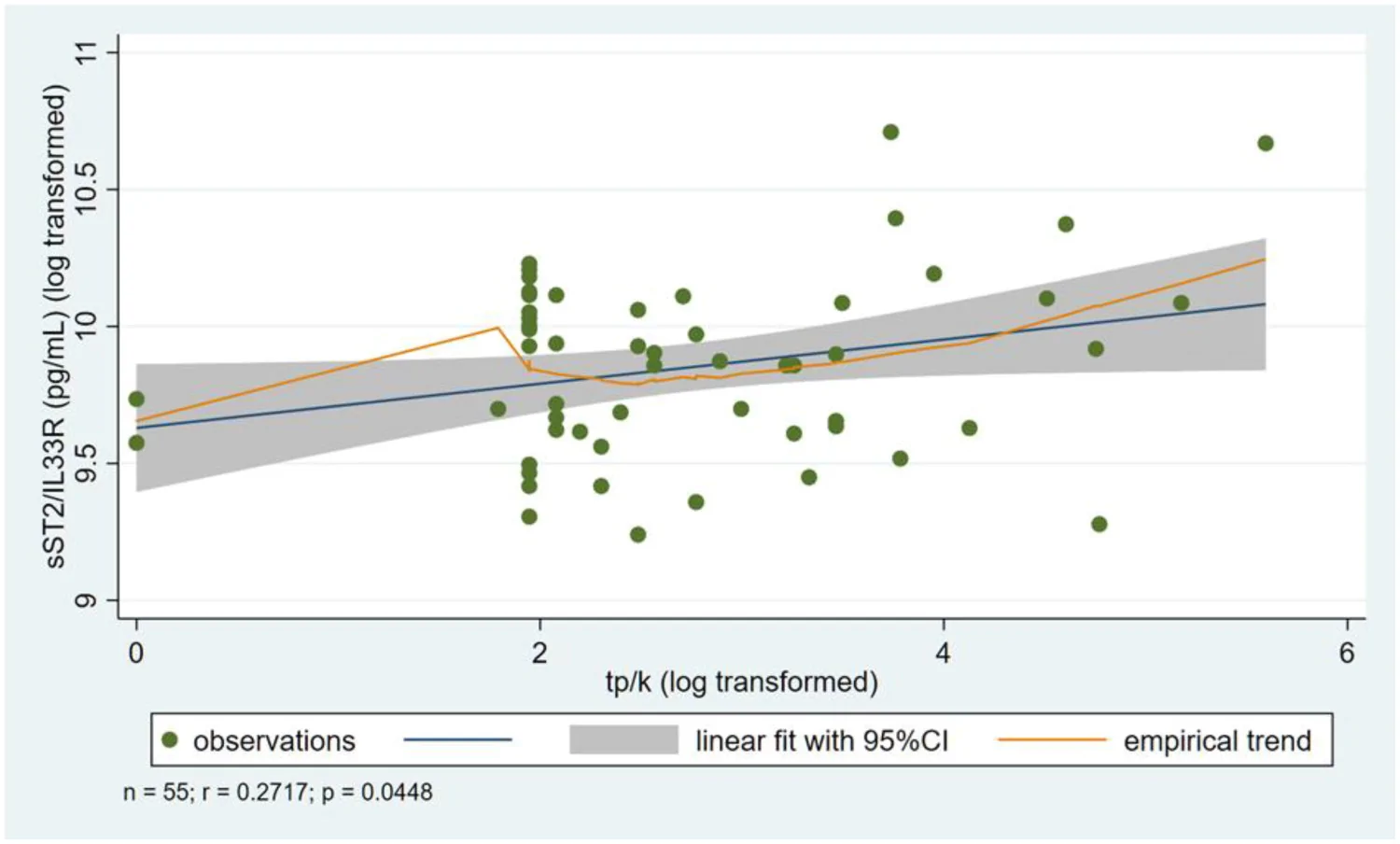
The relationship between sST2/IL33R (pg/mL, log-transformed) and tp/k (log-transformed). This scatterplot shows the relationship between sST2/IL33R (pg/mL, log-transformed) and tp/k (log-transformed), where tp/k likely represents a parameter related to protein clearance or kidney function. Green dots: Individual data points (n = 55). Blue line: Linear regression fit showing the general association between the two variables. Gray band: 95% confidence interval (CI) around the linear fit – reflecting uncertainty in the estimate. Orange line: Empirical (nonlinear smoothed) trend showing the actual shape of the relationship in the data. Statistics below: *r* = 0.2717: Indicates a moderate positive correlation between sST2/IL33R and tp/k. *p* = 0.0448: Statistically significant (*p* < 0.05), suggesting the correlation is unlikely due to random variation.

## Discussion

The onset of proteinuria in renal allograft recipients is frequently associated with an increased risk for both graft failure and mortality. Proteinuria is fairly common immediately after transplantation, and may originate from the native kidney or the allograft, making differential diagnosis difficult [24]. On the other hand, the persistence or late occurrence of proteinuria is indicative of graft injury. The prevalence of proteinuria at 1 year after renal transplantation ranges from 11% to 45%. Several factors can contribute to the pathogenesis of post-transplant proteinuria [1–5]. Roodnat et al. found that new onset proteinuria occurred more frequently in transplant recipients with glomerulonephritis, systemic disease, or arterial hypertension as the primary disease [6]. Residual proteinuria has multiple causes, including recurrent and newly diagnosed glomerular disease, damage caused by anti-HLA class II antibodies, post-transplant glomerular disease, interstitial fibrosis, graft tubular atrophy, and adverse effects of mTOR inhibitor drugs [3–5]. The major focus of CKD treatment has now shifted to preservation of renal function through the use of multifactorial therapy that aims to prevent not only end-stage renal disease but also cardiovascular events, which are major complications of CKD.

Soluble ST2 is a circulating biomarker from the IL-33/ST2 signaling pathway. It reflects myocardial stress, inflammation, and fibrosis. Unlike BNP/NT-proBNP, sST2 is minimally affected by kidney function, which makes it especially useful in patients with CKD and kidney transplants. Our measurement suggests that higher proteinuria (tp/k) is associated with higher circulating sST2, a biomarker of inflammation, myocardial stress, and fibrosis. According to our results, a moderate, but statistically significant, positive correlation was observed between proteinuria and sST2 in kidney transplant recipients with stable graft function.

This relationship supports the hypothesis that renal injury severity may be linked to systemic inflammatory or cardiovascular risk pathways in kidney transplant recipients. Despite statistical significance, the modest correlation indicates that tp/k accounts for only a small proportion of sST2 variability, suggesting that additional factors contribute to sST2 levels.

The Il33 receptor level was found to be independent of GGT, LDH and eGFR values, but not of proteinuria. The malondialdehyde value depends on renal function, but not on hemoglobin, proteinuria, or LDH values. Neprilysin is significantly dependent on GGT but not on eGFR, HGB, or LDH. ACE2 in heart failure kidney transplant recipients depends on eGFR, not on protein excretion, but when the analysis was extended to the entire study population, we did not find a dependence on eGFR. ACE2 activity increases progressively across the range of eGFR. The empirical trend closely follows the linear regression, suggesting that the relationship is largely linear with minimal deviation. Data dispersion is relatively uniform across eGFR values, with slightly wider confidence intervals at the extremes, reflecting fewer observations. Our results suggest that better graft function is associated with higher ACE2 activity in kidney transplant recipients. Given ACE2’s role in counterregulating the renin–angiotensin system, this association may reflect a protective cardiovascular and renal phenotype in patients with preserved graft function. The strength and significance of the association indicate that graft function is an important determinant of circulating ACE2 activity in this population.

The small increase in the number of elevations is a limitation of the study. When analyzing the entire population, no correlation was observed between proteinuria and biomarker levels. However, if we analyze only kidney transplant recipients with heart failure, the correlations are visible, but we did not draw any far-reaching conclusions from the small increase in the number of elevations. We did not examine time since transplantation, which, according to the literature, can modify ACE2 levels, shifting the balance toward chymase activity. In our study, we analyzed only blood measurements, not histological examinations. In the immunosuppressed state, many factors influence the levels of these sensitive parameters. We did not correlate the results with the basic therapy of heart failure. According to the published literature, treating kidney transplant patients with chymase inhibitors reduced the degree of fibrosis in the kidney graft without altering proteinuria.

Because of the small sample size, descriptive statistics have been used, which may compromise the robustness of the findings, as they cannot establish causality and are sensitive to outliers. Therefore, the results should be interpreted as exploratory rather than definitive. Future studies with larger, prospectively collected cohorts will be essential.

## Conclusion

Pharmacological RAS blockade constitutes the cornerstone of antihypertensive therapy in patients with CKD. Although ACE is the predominant Ang II–forming enzyme in healthy kidneys, chymase directs Ang II generation, particularly in aged allografts (> 2 years) (25) The shift from ACE to chymase occurs during the first 2 years after KT. This shift in kidney RAS activity was reflected by the high count of chymase-containing mast cells in long-term kidney transplants. The observed high proportion of chymase-dependent Ang II production in kidney allografts suggests that chymase may be a promising therapeutic target to dampen the progression of allograft fibrosis and its deleterious effects on allograft function and survival. The IL-33/ST2/ILC2 axis represents a critical immunoregulatory pathway in kidney diseases and a critical bridge between epithelial barrier damage (innate danger signals) and type 2 immune-mediated tissue repair or fibrosis. In modern nephrology and cardiology, this pathway is viewed as a double-edged sword that shifts from an acute protective repair mechanism to a chronic driver of organ failure [29].

Kidney-resident ILC2s, localized primarily in the perivascular tubulointerstitium, promote anti-inflammatory and antifibrotic responses through IL-13 production and M2 macrophage polarization. Accumulating evidence from AKI, CKD, and glomerulonephritis models suggests that exogenous IL-33 administration can restore ILC2 function, reduce inflammation, and mitigate fibrosis. However, the effects of IL-33 are highly context-dependent, influenced by dosage, timing, and disease stage – underscoring the need for precise therapeutic modulation. Proteinuria was weakly positively associated with circulating sST2 concentrations in kidney transplant recipients, suggesting a link between renal injury, inflammation, and cardiovascular remodeling. Further studies are needed to clarify the prognostic significance of this pathway in kidney transplant outcomes.

## Data Availability

The data that support the findings of this study are available from the corresponding author upon reasonable request.

## References

[1] Ibis A, Akgül A, Ozdemir N, et al. Posttransplant proteinuria is associated with a higher risk of cardiovascular disease and graft failure in renal transplant patients. Transplant Proc. 2009;41(5):1604–1608. doi: 10.1016/j.transproceed.2008.12.034. 19545689

[2] Diena D, Messina M, DeBiase C, et al. Relationship between early proteinuria and long-term outcome of kidney transplanted patients from different decades of donorage. BMCNephrology. 2019;20:443. 10.1186/s12882-019-1635-0 PMC6889703 31791270

[3] Matsushita K, Coresh J, Sang Y, et al. Estimated glomerular filtration rate and albuminuria for prediction of cardiovascular outcomes: a collaborative meta-analysis of individual participant data. Lancet Diabetes Endocrinol. 2015;3(7):514–525. doi: 10.1016/S2213-8587(15)00040-6. PMC4594193 26028594

[4] Naesens M, Lerut E, Emonds M-P, et al. Proteinuria as a noninvasive marker for renal allograft histology and failure: an observational cohort study. J Am Soc Nephrol. 2016;27(1):281–292. doi: 10.1681/ASN.2015010062. PMC4696583 26152270

[5] Halimi J-M, Laouad I, Buchler M, et al. Early low-grade proteinuria: causes, short-term evolution and long-term consequences in renal transplantation. Am J Transplant. 2005;5(9):2281–2288. doi: 10.1111/j.1600-6143.2005.01020.x. 16095510

[6] Swanson KJ, Aziz F, Garg N, et al. The role of cardiac biomarkers in kidney transplant recipients: role of novel biomarkers in kidney transplantation. World J Transplant. 2020;10(9):230–255. doi: 10.5500/wjt.v10.i9.230. PMC7504189 32995319

[7] Foster MC, Weiner DE, Bostom AG, et al. Filtration markers, cardiovascular disease, mortality, and kidney outcomes in stable kidney transplant recipients: the FAVORIT trial. Am J Transplant. 2017;17(9):2390–2399. doi: 10.1111/ajt.14258. PMC5573607 28257169

[8] Bansal N, Carpenter MA, Weiner DE, et al. Urine injury biomarkers and risk of adverse outcomes in recipients of prevalent kidney transplants: the folic acid for vascular outcome reduction in transplantation trial. J Am Soc Nephrol. 2016;27(7):2109–2121. doi: 10.1681/ASN.2015030292. PMC4926968 26538631

[9] Park M, Katz R, Shlipak MG, et al. Urinary markers of fibrosis and risk of cardiovascular events and death in kidney transplant recipients: the FAVORIT trial. Am J Transplant. 2017;17(10):2640–2649. doi: 10.1111/ajt.14284. PMC5620109 28371433

[10] Devine PA, Cardwell C, Maxwell AP. Association of soluble ST2 with all-cause and cardiovascular mortality in renal transplant recipients: a single-centre cohort study. BMC Nephrol. 2020;21(1):22. doi: 10.1186/s12882-020-1690-6. PMC6986045 31992225

[11] Yepes-Calderón M, Sotomayor CG, Gans ROB, et al. Post-transplantation plasma malondialdehyde is associated with cardiovascular mortality in renal transplant recipients: a prospective cohort study. Nephrol Dial Transplant. 2020;35(3):512–519. doi: 10.1093/ndt/gfz288. PMC7056950 32133530

[12] Kovarik JJ, Shoumariyeh T, Domenig O, et al. Fulacimstat reduces angiotensin II in kidney allografts in a cross-sectional exploratory study. Kidney Int Rep. 2026;11(3):103745. doi: 10.1016/j.ekir.2025.103745. PMC12857369 41624455

[13] Ráduly AP, Nagy L, Bódi B, et al. Biomarkers in heart failure: traditional and emerging indicators for prognosis. ESC Heart Fail. 2025;12(3):1535–1538. doi: 10.1002/ehf2.15168. PMC12055335 39545304

[14] Bozkurt B, Nair AP, Misra A, et al. Neprilysin inhibitors in heart failure: the science, mechanism of action, clinical studies, and unanswered questions. JACC Basic Transl Sci. 2023;8(1):88–105. doi: 10.1016/j.jacbts.2022.05.010. PMC9911324 36777165

[15] Pavo N, Arfsten H, Cho A, et al. The circulating form of neprilysin is not a general biomarker for overall survival in treatment-naïve cancer patients. Sci Rep. 2019;9(1):2554. doi: 10.1038/s41598-019-38867-2. PMC6385211 30796257

[16] Barksby HE, Lea SR, Preshaw PM, et al. The expanding family of interleukin-1 cytokines and their role in destructive inflammatory disorders. Clin Exp Immunol. 2007;149(2):217–225. doi: 10.1111/j.1365-2249.2007.03441.x. PMC1941943 17590166

[17] Schmitz J, Owyang A, Oldham E, et al. IL-33, an interleukin-1-like cytokine that signals via the IL-1 receptor-related protein ST2 and induces T helper type 2-associated cytokines. Immunity. 2005;23(5):479–490. doi: 10.1016/j.immuni.2005.09.015. 16286016

[18] Lécart S, Lecointe N, Subramaniam A, et al. Activated, but not resting human Th2 cells, in contrast to Th1 and T regulatory cells, produce soluble ST2 and express low levels of ST2L at the cell surface. Eur J Immunol. 2002;32(10):2979–2987. doi: 10.1002/1521-4141(2002010)32:10<2979::AID-IMMU2979>3.0.CO;2-5. 12355452

[19] Brint EK, Xu D, Liu H, et al. ST2 is an inhibitor of interleukin 1 receptor and Toll-like receptor 4 signalling and maintains endotoxin tolerance. Nat Immunol. 2004;5(4):373–379. doi: 10.1038/ni1050. 15004556

[20] Weinberg EO, Shimpo M, De Keulenaer GW, et al. Expression and regulation of ST2, an interleukin-1 receptor family member, in cardiomyocytes and myocardial infarction. Circulation. 2002;106(23):2961–2966. doi: 10.1161/01.cir.0000038705.69871.d9. PMC1460012 12460879

[21] Xu D, Chan WL, Leung BP, et al. Liew FY elective expression of a stable cell surface molecule on type 2 but not type 1 helper T cells. J Exp Med. 1998;187(5):787–794. doi: 10.1084/jem.187.5.787. PMC2212173 9480988

[22] Löhning M, Stroehmann A, Coyle AJ, et al. T1/ST2 is preferentially expressed on murine Th2 cells, independent of interleukin 4, interleukin 5, and interleukin 10, and important for Th2 effector function. Proc Natl Acad Sci U S A. 1998;95(12):6930–6935. doi: 10.1073/pnas.95.12.6930. PMC22690 9618516

[23] Fagyas M, Fejes Z, Sütő R, et al. Circulating ACE2 activity predicts mortality and disease severity in hospitalised COVID-19 patients. Int J Infect Dis. 2022;115:8–16. doi: 10.1016/j.ijid.2021.11.028. PMC8613979 34838959

[24] Hashmi AJ, Bujaki B, Bidiga L, et al. Relevance of proteinuria in kidney transplant recipients and allograft outcomes. Transplant Proc. 2024;56:1273–1279. 10.1016/j.transproceed.2024.06.002 39034194

[25] Pethő ÁG, Tapolyai M, Browne M, et al. The importance of the nephrologist in the treatment of the diuretic-resistant heart failure. Life (Basel). 2023;13(6):1328. doi: 10.3390/life13061328. PMC10303045 37374112

[26] Kakkar R, Lee RT. The IL-33/ST2 pathway: therapeutic target and novel biomarker. Nat Rev Drug Discov. 2008;7(10):827–840. doi: 10.1038/nrd2660. PMC4277436 18827826

[27] Takai S, Jin D, Muramatsu M, et al. Therapeutic applications of chymase inhibitors in cardiovascular diseases and fibrosis. Eur J Pharmacol. 2004;501(1-3):1–8. doi: 10.1016/j.ejphar.2004.08.040. 15464056

[28] Nagy B. Jr, Fejes Z, Szentkereszty Z, et al. A dramatic rise in serum ACE2 activity in a critically ill COVID-19 patient. Int J Infect Dis. 2021;103:412–414. doi: 10.1016/j.ijid.2020.11.184. PMC7689308 33249290

[29] Matsuzaka Y, Iyoda M. The IL-33/ST2/ILC2 pathway in kidney disease: balancing inflammation, fibrosis, and repair. Am J Physiol Cell Physiol. 2025;329(3):C718–C725. doi: 10.1152/ajpcell.00405.2025. 40707033

